# Alpha-1 Antitrypsin Augmentation Inhibits Proteolysis of Neutrophil Membrane Voltage-Gated Proton Channel-1 in Alpha-1 Deficient Individuals

**DOI:** 10.3390/medicina57080814

**Published:** 2021-08-10

**Authors:** Padraig Hawkins, Julian Sya, Nee Kee Hup, Mark P. Murphy, Noel G. McElvaney, Emer P. Reeves

**Affiliations:** Irish Centre for Genetic Lung Disease, Department of Medicine, Royal College of Surgeons in Ireland, Beaumont Hospital, D02 YN77 Dublin, Ireland; padraighawkins@rcsi.ie (P.H.); juliansya@rcsi.ie (J.S.); NeeKeeHup@rcsi.ie (N.K.H.); MMurphy@rcsi.ie (M.P.M.); gmcelvaney@rcsi.ie (N.G.M.)

**Keywords:** alpha-1 antitrypsin, neutrophils, elastase, voltage-gated proton channel-1

## Abstract

*Background and Objectives*: Alpha-1 antitrypsin is a serine protease inhibitor that demonstrates an array of immunomodulatory functions. Individuals with the genetic condition of alpha-1 antitrypsin deficiency (AATD) are at increased risk of early onset emphysematous lung disease. This lung disease is partly driven by neutrophil mediated lung destruction in an environment of low AAT. As peripheral neutrophil hyper-responsiveness in AATD leads to excessive degranulation and increased migration to the airways, we examined the expression of the membrane voltage-gated proton channel-1 (HVCN1), which is integrally linked to neutrophil function. The objectives of this study were to evaluate altered HVCN1 in AATD neutrophils, serine protease-dependent degradation of HVCN1, and to investigate the ability of serum AAT to control HVCN1 expression. *Materials and Methods*: Circulating neutrophils were purified from AATD patients (*n* = 20), AATD patients receiving AAT augmentation therapy (*n* = 3) and healthy controls (*n* = 20). HVCN1 neutrophil expression was assessed by flow cytometry and Western blot analysis. Neutrophil membrane bound elastase was measured by fluorescence resonance energy transfer. *Results*: In this study we demonstrated that HVCN1 protein is under-expressed in AATD neutrophils (*p* = 0.02), suggesting a link between reduced HVCN1 expression and AAT deficiency. We have demonstrated that HVCN1 undergoes significant proteolytic degradation in activated neutrophils (*p* < 0.0001), primarily due to neutrophil elastase activity (*p* = 0.0004). In addition, the treatment of AATD individuals with AAT augmentation therapy increased neutrophil plasma membrane HVCN1 expression (*p* = 0.01). *Conclusions*: Our results demonstrate reduced levels of HVCN1 in peripheral blood neutrophils that may influence the neutrophil-dominated immune response in the AATD airways and highlights the role of antiprotease treatment and specifically AAT augmentation therapy in protecting neutrophil membrane expression of HVCN1.

## 1. Introduction

Alpha-1 antitrypsin (AAT) is the archetypal member of the serine protease inhibitor (serpin) superfamily [1,2]. AAT is delivered in an inducible manner from the liver and blood cells upon activation of serine protein inhibitor-A1 (SERPINA1) inflammation-responsive promoter [3]. AAT is most abundant in plasma at a concentration of approximately 1.5 g/L. Moreover, AAT protein levels can increase secondary to acute or chronic inflammatory states, as well as rise in normal physiological conditions, such as pregnancy [4]. From the circulation, AAT passes into many body compartments [5], and concentrations of AAT in lung epithelial lining fluid are approximately 10% of that of the circulation [6]. AAT can also be secreted locally by structural and immune cells of the airways including lung epithelial cells, macrophages, and neutrophils [7]. The detailed structure of AAT is optimal for serine protease activity inhibition, most notably neutrophil elastase (NE), and exploits a short reactive center loop (RCL, positions 357–366) as a sequence-specific bait. Of importance, cigarette smoke can cause oxidation of met 358 and met 351 of the RCL, and has been shown to reduce the NE inhibiting capacity of AAT dramatically [8]. The involvement of individuals with AAT deficiency (AATD) in research studies has been instrumental in delineating the anti-protease activity, and the more recently assigned immune-regulatory functions of AAT [9,10,11,12,13,14,15,16].

AATD is an autosomal co-dominant condition caused by mutations in SERPINA1 [17]. AAT expression is influenced by the codominant expression of two SERPINA1 gene alleles. The most common alleles comprise: (i) the normal M; (ii) the S (Glu264Val) which is associated with moderately reduced AAT plasma levels; (iii) the severely deficient Z (Glu342Lys) with AAT levels of ~10–15% of normal [18,19,20]. MM genotype class encompasses a variety of normal M genetic variants, while the remaining classes comprise different combinations of variants, including MS, SS, MZ and ZZ, which correspond to different mild to severe forms of AATD. Z and S are therefore pathogenic mutations which have been linked to AATD [17]. Clinical presentation of AATD includes early onset lung emphysema identified by computed tomography [21], particularly in AATD patients who smoke [22]. Interestingly, the diagnosis of AATD with increasing severity of genotype is associated with lower current-smoking rates among ever-smokers [23]. Of importance however, neutrophilia is a feature of AATD airways disease, with increased neutrophil numbers reported in the epithelial lining fluid of nonsmoking AATD individuals, resulting in unrestricted neutrophil elastolytic activity [24]. In turn, NE itself can amplify the cycle of inflammation in AATD by stimulating the production of immune cell chemotactic factors, including leukotriene B_4_, by AATD alveolar macrophages [24] and neutrophils [25], and the production of the potent chemokine interleukin-8 by epithelial cells [26]. Moreover, neutrophil receptors can be affected by high protease activity [27,28] and NE cleavage of protease-activated receptor 2 leads to excessive extracellular reactive oxygen species (ROS) production in the absence of AAT [29]. AAT augmentation therapy is the only specific therapy approved by the Food and Drug Administration for the treatment of AATD, demonstrating a reduced rate of lung density loss [30,31], and alleviation of the described NE neutrophil signaling effects [25,29].

Due to the reported functional changes in AATD neutrophils, including increased chemotaxis [32], increased primary granule degranulation [25], delayed bacterial killing [33], and a recent study demonstrating alterations in the membrane proteome of circulating AATD neutrophils [29], we chose to explore the expression of membrane voltage-gated proton channel-1 (HVCN1). HVCN1 activity is integrally linked to NADPH-oxidase activity of neutrophils and functions to transfer protons from the cytosol to the vacuolar lumen or extracellular milieu, thereby preventing membrane depolarization. Altered HVCN1 activity or expression results in reduced oxidase activity and impaired microbial killing [34], yet increased neutrophil primary granule release [35]. Thus, the aim of this study was to perform the first analysis of plasma membranes of neutrophils from AATD patients for HVCN1 expression, and to identify proteases involved in altered membrane protein expression of this proton channel. Finally, our objective was to elucidate the impact of AAT augmentation therapy on HVCN1 expression.

## 2. Materials and Methods

### 2.1. Chemicals and Reagents

All chemicals and reagents used in this study were of the highest purity available and endotoxin-free and were purchased from Sigma-Aldrich (Dublin, Ireland) unless otherwise indicated.

### 2.2. Study Design

Healthy nonsmoking control subjects were recruited (*n* = 20) and were defined as having an MM-AAT phenotype by isoelectric focusing with serum AAT concentrations within the normal range (25–5 μM) and showing no evidence of any inflammatory or lung disease. Nonsmoking PI*ZZ patients (*n* = 20, FEV1 (% predicted 60.52 ± 3.86)) were recruited from the Irish Alpha-1 Antitrypsin Deficiency outpatient clinic in Beaumont Hospital. PI*ZZ patients receiving augmentation therapy (CSL Behring, 60 mg/kg IV weekly) on day 0 and day 2 of treatment were recruited as previously described [32]. All AATD subjects were free from pulmonary exacerbation in the eight weeks prior to enrolment to the study. All participants provided written informed consent.

### 2.3. Neutrophil Isolation and Assays

Human neutrophils were isolated from heparinized (10 U/mL; Sarstedt, Numbrecht, Germany) venous blood as previously described [36]. Cells were resuspended in phosphate buffered saline (PBS) containing 5mM glucose (PBSG). The purity of the neutrophil population was confirmed by flow cytometry measuring the neutrophil membrane marker CD16b. Cell purity was found to be >99% [32]. Neutrophil viability was systematically monitored before and after each treatment by Trypan Blue exclusion or by MTT (3-(4,5-dimethylthiazol-2-yl)-2,5-diphenyl tetrazolium bromide) assay and found to be >98%. Cells remained unstimulated or stimulated with a combination of TNFα/fMLP (10 ng/10 µM/10^7^ cells) prior to processing. In a subset of experiments, the protective effect of protease inhibitors on HVCN1 levels was assessed by stimulating cells in the presence of pefabloc (1 mM), E-64 (10 µM) or EDTA (5 mM). To examine the effects of exogenous NE on HVCN1 levels, 20nM of purified human sputum elastase (Elastin Products) was incubated with 2 × 10^6^ neutrophils at 37 °C and aliquots were removed at 0, 10, 30 and 60 min. Subsequently, HVCN1 levels were analyzed by flow cytometry.

Activity of neutrophil membrane bound NE was measured by fluorescence resonance energy transfer (FRET). An NE specific FRET substrate was used (Abz-Ala-Pro-Glu-Glu-Ile-Met-Arg-Arg-Gln-EDDnp, 3230-v, Peptide Institute, Inc, Osaka, Japan). The FRET substrate was reconstituted in 30% (*w*/*v*) dimethylformamide (DMF) to give a concentration of 1mM. Neutrophils (1 × 10^6^ cells/mL) were isolated from AATD individuals and healthy controls and were activated with TNF-α/fMLP by incubation at 37 °C for up to 60 min and were then added to the wells of a 96 well plate in PBS (150 µL). Reactions commenced with the addition of 3 μL of the 1 mM working substrate solution and fluorescence was recorded at excitation 320 nm and emission 420 nm, as previously described [25].

### 2.4. SDS-Polyacrylamide Gel Electrophoresis and Western Blotting

Neutrophil (1 × 10^7^) whole cell lysates from healthy control donors or AATD patients were prepared in lysis buffer (150 mM NaCl, 50 mM Tris HCl, 1% (*v*/*v*) Triton X-100, 0.2 mM NaVO_4_, 1 mM DTT, 10 mM NaF, 1 mM EDTA and protease inhibitors) and processed as previous described [37]. For SDS-PAGE analysis electrophoresis of samples was conducted according to Laemmli’s method [38]. Denatured protein samples (20 μL) were resolved on 10% (*w*/*v*) resolving gel and 4% (*w*/*v*) stacking gel. SeeBlue Plus2 Prestained molecular mass markers (4 µL; Invitrogen) were loaded on each gel for determination of molecular weight. Gels were run in an ATTO AE6450 electrophoresis tank (ATTO Corporation, Tokyo, Japan) and electrophoresis was carried out for 60–90 min at 150 V.

Following electrophoresis, proteins were transferred onto PVDF membrane at 150 mA for 60 min using wet transfer blotting apparatus. Following transfer, membranes were blocked with 5% (*w*/*v*) nonfat powdered milk in PBS containing 0.1% (*v*/*v*) Tween-20 (PBST) for 1 h at room temperature. For immunological detection of HVCN1 1 μg/mL rabbit anti-HVCN1 antibody (Sigma, SAB3500536) followed by HRP-linked goat anti-rabbit IgG secondary antibody was employed. As a loading control, immunoblots were probed for actin using 1 μg/mL mouse anti-actin (Santa Cruz Biotechnology, Heidelberg, Germany). Immunoreactivity was detected using Immobilon Western Chemiluminescent HRP substrate (Millipore) solution and a G-Box Chemie XL (Syngene) and analyzed using GeneSnap and GeneTools software.

### 2.5. Flow Cytometry Experiments

Neutrophils were fixed (4% (*w*/*v*) paraformaldehyde) and blocked (2% (*w*/*v*) BSA) for 30 min at room temperature. After washing (PBS × 2) neutrophils (1 × 10^6^) were incubated with 1 μg/100 μL of mouse monoclonal anti-CD16b (Santa Cruz, Heidelberg, Germany) for determining neutrophil purity. Control samples were exposed to relevant nonspecific isotype control IgG or secondary labelled antibody alone (FITC labelled bovine anti-mouse; Santa Cruz Biotechnology). For whole cell plasma membrane levels of HVCN1, samples were fixed, blocked and probed with a rabbit anti-HVCN1 antibody (Sigma, SAB3500536) for 1 h followed by incubation with an anti-rabbit FITC labelled IgG secondary antibody (Abcam, ab6717) and analyzed by flow cytometry. FITC Goat anti-Rabbit IgG served as a control. Samples were analyzed on a FACScalibur^TM^ flow cytometer (Becton Dickinson, San Jose, CA, USA). At least 10,000 events were acquired and the mean fluorescence intensity for each experiment was determined using BD CellQuest Pro software or FlowJo software.

### 2.6. Statistical Analysis

Results are expressed as mean ± standard error of the mean (SEM) of n separate biological replicates as stated in the figure legends. Statistical analysis was performed with GraphPad Prism (version 4.03 for Windows). For statistical comparison of small datasets (*n* < 6) Student’s t test was performed to determine *p* values [39]. For larger datasets the D’Agostino and Pearson omnibus normality test was carried out to determine whether data was normally distributed. When normally distributed, groups were compared by Student’s *t* test, otherwise by the nonparametric Mann−Whitney U test. For comparison of three or more groups one-way ANOVA was performed. *p* values were considered statistically significant with *p* < 0.05.

## 3. Results

### 3.1. Cell Membrane Expression of HVCN1 in Neutrophils of AATD Is Decreased

**Figure 1:**
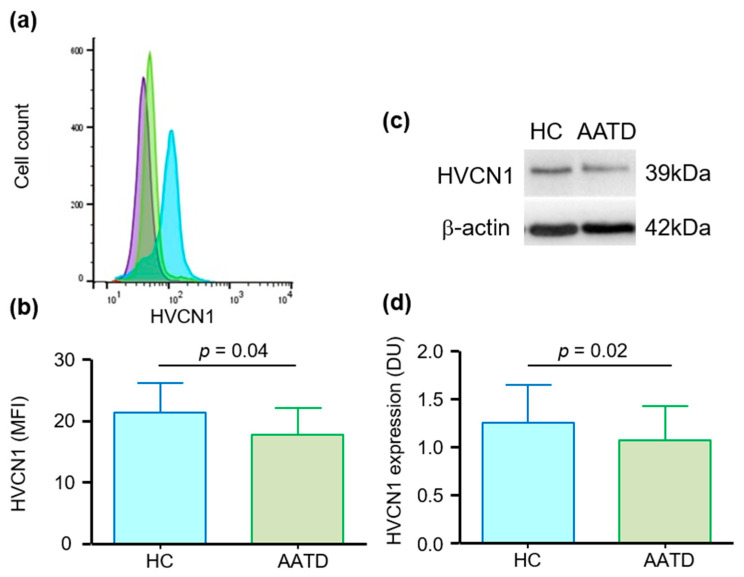
Reduced voltage-gated hydrogen channel 1 (HVCN1) in neutrophils of individuals with alpha-1 antitrypsin deficiency (AATD). (**a**) Representative flow cytometry data histogram showing abundance of cell surface HVCN1 in healthy control (HC) (blue) and AATD (green) neutrophils compared to the isotype control (purple). (**b**) Neutrophil membrane levels of HVCN1 quantified by flow cytometry were expressed as mean fluorescence intensity (MFI). Graph illustrates a significantly lower abundance of HVCN1 in AATD neutrophils (*n* = 9 subjects per group, *p* = 0.04, Student’s *t* test). (**c**) Representative Western blot depicting HVCN1 expression in neutrophil whole cell lysates. β-actin was used as a loading control. (**d**) Graph depicting densitometry units (DU) for HVCN1 immunobands, showing significantly decreased protein level of HVCN1 in neutrophil whole cell lysates of individuals with AATD compared to HC (*n* = 7 subjects per group, *p* = 0.02, Student’s *t* test).

As HVCN1 is localized to neutrophil plasma membranes [40], the experiments measured the abundance of HVCN1 on the surface of circulating neutrophils by flow cytometry (Figure 1a). Although variable across nine subjects, results revealed a significant 20% reduction in the abundance of HVCN1 on the outer plasma membrane surface of AATD neutrophils (*p* = 0.04) (Figure 1b). HVCN1 in AATD neutrophils was also assessed by Western blotting (Figure 1c). The abundance of HVCN1 was quantified by densitometric analysis of immunobands with the results demonstrating the abundance of HVCN1 in AATD neutrophil whole cell lysates to be significantly reduced by 15% compared to HC cell fractions (*p* = 0.02) (Figure 1d). Collectively, these results demonstrate a reduced abundance of HVCN1 in circulating AATD neutrophils. The reduction was of the magnitude of 15–20%. Ensuing experiments investigated the cause for lower abundance of HVCN1 in AATD cells.

### 3.2. HVCN1 Is Proteolytically Cleaved from Neutrophil Plasma Membranes in AATD

**Figure 2:**
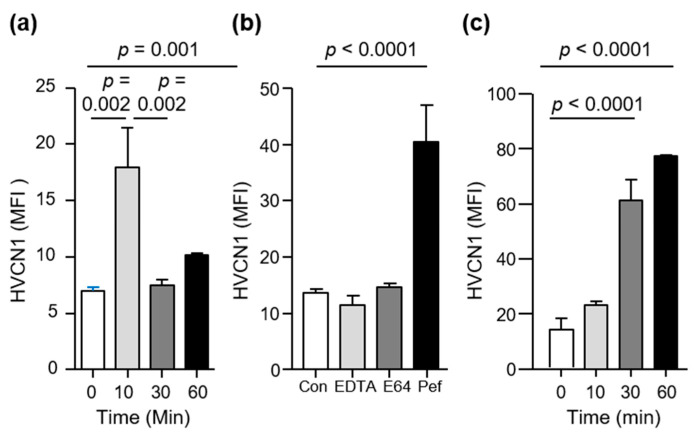
HVCN1 is reduced on activated neutrophil plasma membranes by serine proteases. (**a**) Time course of TNF-α/fMLP (10 ng/10 μM) neutrophil activation with HVCN1 membrane expression assessed by flow cytometry. Significantly increased abundance of HVCN1 at 10 min compared to 0 min (*p* = 0.001, *n* = 6, one-way ANOVA, post hoc Tukeys’ test). (**b**) TNF-α/fMLP activation of neutrophils for 30 min (Con) in the presence of nonspecific protease inhibitors EDTA, E64 or pefabloc (Pef). Pef treatment resulted in significantly increased abundance of HVCN1 (*p* < 0.0001, *n* = 3, one-way ANOVA, post hoc Dunnetts’ test). (**c**) Time course of neutrophil activation in the presence of Pef. Inclusion of Pef resulted in increased levels of HVCN1 (*p* < 0.0001, *n* = 6 technical repeats, one-way ANOVA, post hoc Tukeys’ test).

The inflammatory induced changes to the membrane proteome of circulating AATD neutrophils has been previously described [29]. Accordingly, studies were conducted to determine whether neutrophil exposure to inflammatory stimuli over a prolonged period of time could influence HVCN1 plasma membrane expression. Cells were activated with the soluble stimuli TNF-α (10 ng/2 × 10^7^ cells) in combination with fMLP (10 μM) and HVCN1 expression was monitored by flow cytometry (Figure 2). Cell activation induced a significant increase in HVCN1 expression on plasma membranes after 10 min (*p* = 0.002), but after 30 min a reduction in HVCN1 levels had commenced (*p* = 0.002) (Figure 2a). As TNF-α/fMLP stimulation can cause degranulation and the release of proteolytic enzymes to the outside of the cell [29,41,42], a systematic approach was adopted to investigate the possible cleavage of HVCN1 from plasma membranes and to categorize the proteases involved. Nonspecific protease inhibitors targeting the main protease families were employed including E64, an inhibitor of cysteine proteases, EDTA a general metalloprotease inhibitor, and pefabloc a potent serine protease inhibitor [43]. After 30 min cell activation only pefabloc (1 mM) prevented a reduction in HVCN1 membrane expression (*p* < 0.0001) (Figure 2b). Moreover, while pefabloc maintained the expression of HVCN1, E-64 and EDTA had no protective effect on the surface expression of HVCN1.This set of experiments was extended to include a time course of TNF-α/fMLP neutrophil activation in the presence of pefabloc, with significantly increased abundance of HVCN1 recorded at 30 (*p* < 0.0001) and 60 min (*p* < 0.0001), confirming a serine protease responsible for HVCN1 cleavage (Figure 2c). These results show that HVCN1 is increased on neutrophil plasma membranes following cell stimulation, but that HVCN1 levels subsequently decrease following prolonged activation. In subsequent experiments, the serine protease responsible for HVCN1 depletion from the outer plasma membrane was investigated.

### 3.3. HVCN1 Is Proteolytically Cleaved by Neutrophil Elastase

**Figure 3:**
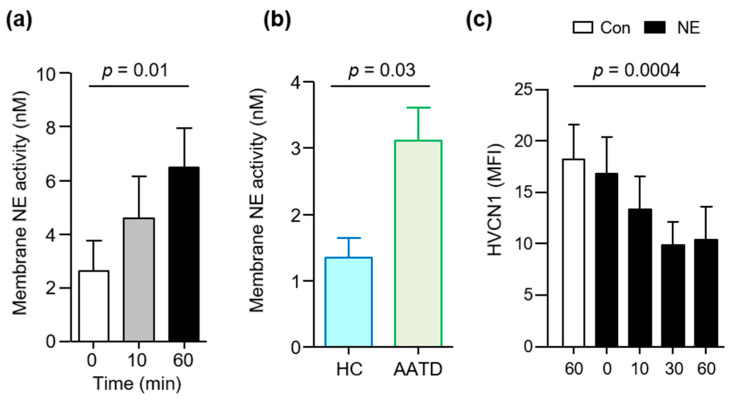
NE activity on AATD neutrophil membranes cleaves HVCN1. (**a**) Significantly increased NE activity on healthy control (HC) neutrophil plasma membranes after TNF-α (10 ng)/fMLP (10 μM) stimulation (*n* = 5 biological repeats, as measured by FRET assay (*p* = 0.01, one-way ANOVA, post hoc Bonferroni test)). (**b**) Activity of NE on unstimulated HC or AATD neutrophil plasma membranes. AATD neutrophils exhibit significantly increased activity of NE compared to HC (*n* = 3 subjects per group, *p* = 0.03, Student’s t test). (**c**) HC neutrophils (2 × 10^6^) were untreated (Con) or exposed to NE (20 nM) over a 60 min time course. Exposure to NE significantly reduced membrane expression of HVCN1 after 60 min (*p* = 0.0004, *n* = 5, one-way ANOVA, post hoc Dunnetts’ test).

The neutrophils of AATD patients degranulate increased levels of primary granule components, including NE [29], and NE rebinding to outer plasma membranes of neutrophils has been reported [25]. Thus, our aim in the following set of experiments was to measure the abundance of NE on neutrophil membranes following exposure to TNF-α and fMLP. Results demonstrated a significant 2.5-fold increase in levels of membrane bound NE activity at 60 min (Figure 3a) (*p* = 0.01). This set of experiments was expanded to compare the activity of NE on neutrophil membranes of unstimulated AATD individuals to that of HC cells. We found that the activity of membrane bound NE on AATD neutrophils was more than double that of HC cells (*p* = 0.03) (Figure 3b).

Ensuing experiments were designed to examine the ability of NE to cleave the neutrophil plasma membrane HVCN1 in vitro. Isolated, circulating HC neutrophils were incubated in the presence or absence of exogenous NE (20 nM). NE cleaved HVCN1 from the surface of purified neutrophils, with a significant 40% reduction observed after 30 min incubation compared to untreated cells (*p* = 0.0004) (Figure 3c). Collectively these results indicate that sustained activity of NE on neutrophil plasma membranes, negatively impacts HVCN1 membrane protein expression.

### 3.4. AAT Augmentation Therapy Increases HVCN1 Expression on AATD Neutrophils

**Figure 4:**
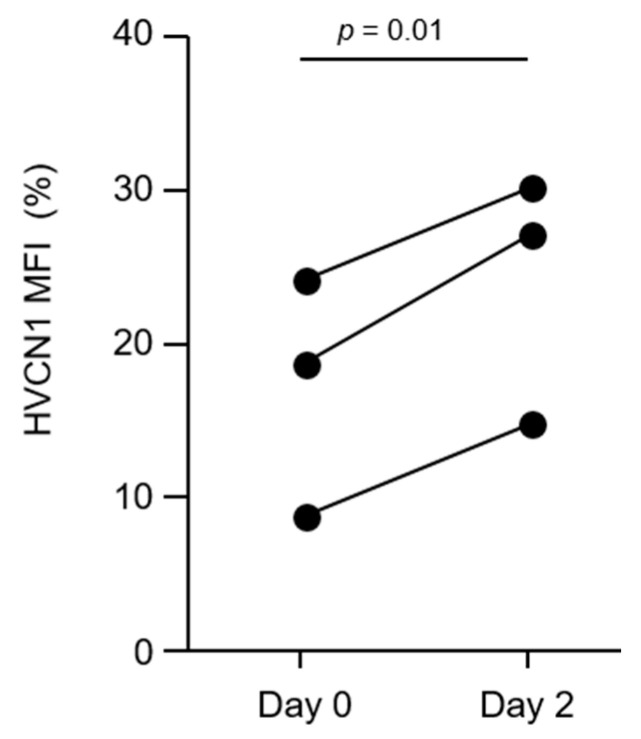
AAT augmentation therapy increases HVCN1 neutrophil plasma membrane expression. HVCN1 membrane expression of AATD neutrophils before (day 0) and 2 days after augmentation therapy (day 2) assessed by flow cytometry. HVCN1 expressing neutrophils were significantly higher on day 2 after therapy (*p* = 0.014, *n* = 3, Student’s paired t test).

We have demonstrated that AATD neutrophils have increased plasma membrane NE activity, which can cleave HVCN1 from the neutrophil surface. To further validate this point, we explored the impact of AAT augmentation therapy administered intravenously each week [31]. Patients with AATD receiving IV AAT replacement therapy were recruited. AATD patients had a blood sample drawn on day 0 (on the day of their infusion, immediately prior to administration) and a second blood sample on day 2, after infusion. This is similar timing of sample collection as previously described [32,42]. Results demonstrate that membrane HVCN1 on AATD neutrophils significantly increased by 40% on day 2, compared to day 0 (*p* = 0.01) (Figure 4). IV replacement therapy has previously been shown to increase plasma levels of AAT to HC levels [33], and to reduce membrane serine protease activity [25,29]. In the current study, we extend these observations and demonstrate that AAT therapy increased plasma membrane levels of HVCN1, a crucial H^+^ transporter linked to neutrophil activity.

## 4. Discussion

HVCN1 is a voltage-gated proton channel, the activity of which supports neutrophil ROS production, a major component of microbial killing. Excess neutrophils and unopposed neutrophil elastase activity underlie the pathogenic imbalance in the lungs of individuals with AATD, more than this, neutrophil function has been shown to be altered in many ways in individuals with AATD [7]. Here we have shown that HVCN1 is cleaved from the surface of neutrophils by NE in vitro. In individuals with AATD, this results in depletion of neutrophil levels of the plasma membrane HVCN1, a proteolytic defect corrected by intravenous AAT therapy in these individuals.

Individuals with severe AATD are at great risk of developing chronic obstructive pulmonary disease (COPD), particularly if there is the additional insult of cigarette smoke exposure. Similar to COPD in AAT sufficient individuals, patients with AATD-COPD display symptoms of cough, sputum production, dyspnea and fatigue [44]. The natural history of AATD-COPD is of accelerated lung function decline, greater than that seen in common COPD not due to AATD [45]. The course of the disease is punctuated by exacerbations, or temporary worsening of symptoms that require a change in therapy, usually oral corticosteroids. Exacerbations are an important metric in both normal COPD and AATD-COPD, and are associated with an accelerated decline in lung function, hospitalization and impaired quality of life. However, while exacerbation frequency in COPD and AATD-COPD are similar, there is some evidence that exacerbations may be of longer duration in individuals with AATD [46,47]. AATD is associated with greater influx of neutrophils to the airways [48,49], but whether the number of recruited cells and associated neutrophil protease activity influences the duration of an exacerbation in AATD is unclear. Of importance however, it has been shown that intravenous augmentation therapy may reduce the occurrence of exacerbations in AATD [50].

In the current study, we demonstrate that circulating neutrophils from individuals with AATD have a reduced abundance of HVCN1 proton channels at the membrane level. This is of importance as HVCN1 has been shown to both promote and inhibit NADPH oxidase activity [51,52,53]. During phagocytosis, when there is a requirement for large quantities of intra-phagosomal ROS, HVCN1 supports oxidase activity for adequate microbial killing. In contrast, under conditions when a relatively small amount of ROS is produced (e.g., exposure to cytokines and chemokines), HVCN1 acts to inhibit ROS production. Consequently, relative to our findings here, in AATD neutrophils lower HVCN1 protein expression may correspond to increased ROS production in response to cytokines and chemokine challenge [52]. In support of this theory, compared to healthy control neutrophils, increased superoxide production by AATD neutrophils was recorded in response to TNF-α or anti-lactoferrin IgG autoantibodies, either employed individually or in combination [42].

The relevance of low levels of HVCN1 is further compounded by research studies demonstrating that neutrophils of *Hvcn1^-/-^* mice demonstrate alkaline phagocytic vacuoles and acidic cytosols following engulfment of microbes, as a consequence of the inability to move protons across the plasma membrane [54]. Phagocytic protease and peroxidase activity is supported by changes in phagosomal pH, governed by HVCN1 activity [51]. Alkalinization of phagocytic vacuoles due to reduced levels of HVCN1 would support serine proteases activity [55], but would be less supportive of enzymes with low pH optima, including myeloperoxidase, azurocidin or α-defensin [41,56,57]. In line with this, killing of *S. aureus* by *Hvcn1^-/-^* bone marrow cells is impaired [51]. Although impaired bacterial killing by AATD neutrophils has been described [33], whether the 20% reduction in levels of HVCN1 recorded in the current study is sufficient to impact phagosomal pH requires further exploration.

Of importance, a further consequence of reduced levels of HVCN1 was reported by Okochi and colleagues, involving the regulation of primary granule degranulation. By employing *Hvcn1^-/-^* mice, these authors demonstrated that neutrophils deficient in HVCN1 released significantly more primary granule components following stimulation, including increased levels of MPO and NE [35]. Intriguingly, they also demonstrated that following fungal lung infection with *Candida albicans*, airway inflammation was more severe in *Hvcn1^-/-^* mice than in wild-type controls, despite being able to efficiently clear the infection. A net increase in production of the potent anti-microbial oxidant hypochlorous acid by *Hvcn1^-/-^* neutrophils was recorded in comparison to wild-type cells, likely related to the excess release of MPO from primary granules. In AATD, it is plausible that lung inflammation may be more severe following microbial infection due to excess primary granule content release in the face of a deficiency of HVCN1. In support of this concept, increased primary granule degranulation by AATD neutrophils in response to leukotriene B_4_ and BLT1 membrane receptor engagement has been demonstrated [25], and was further confirmed in a recent plasma membrane proteomics study, demonstrating increased activation of the Rho GTPase Rac2 and extracellular MPO release [29].

## 5. Conclusions

Our results demonstrate that peripheral blood neutrophils from individuals with AATD have lower levels of the proton channel HVCN1, likely as a result of excess NE and this abnormality is corrected in vivo by AAT augmentation therapy. It is important to acknowledge that our study is limited by the enrollment of small patient numbers. Nevertheless, although observational, it forms a framework for what we consider to be an intriguing question related to the intricate relationship of neutrophil proteases, the voltage-gated proton channel HVCN1 and NAPDH oxidase, with a potential impact on airway inflammation in AATD. Our findings further support the use of intravenous augmentation therapy in individuals with severe AATD and established lung disease.
